# Epidemiology of Atrial Fibrillation and Related Myocardial Ischemia or Arrhythmia Events in Chinese Community Population in 2019

**DOI:** 10.3389/fcvm.2022.821960

**Published:** 2022-04-04

**Authors:** Cheng Li, Haicheng Wang, Mohan Li, Xiangjun Qiu, Qunshan Wang, Jian Sun, Mei Yang, Xiangfei Feng, Shu Meng, Pengpai Zhang, Bo Liu, Wei Li, Mu Chen, Yan Zhao, Rui Zhang, Binfeng Mo, Yuling Zhu, Baohong Zhou, Min Chen, Xia Liu, Yuelin Zhao, Mingzhen Shen, Jinkang Huang, Li Luo, Hong Wu, Yi-Gang Li

**Affiliations:** ^1^Department of Cardiology, Xinhua Hospital, Shanghai Jiao Tong University School of Medicine, Shanghai, China; ^2^Shanghai Siwei Medical Co. Ltd., Shanghai, China; ^3^Medical Information Telemonitoring Center, School of Medicine, Shanghai Jiao Tong University, Shanghai, China; ^4^School of Public Health, Fudan University, Shanghai, China; ^5^Shanghai Municipal Health Commission, Shanghai, China

**Keywords:** atrial fibrillation, prevalence, arrhythmia, myocardial ischemia, China

## Abstract

**Background:**

Atrial fibrillation (AF) is the most common arrhythmia, and the incidence increases rapidly all over the world. The global prevalence of AF (age-adjusted) is 0.60% for men and 0.37% for women and the prevalence of AF in China is 0.65%. It is expected that the number of patients with AF will continue to rise in the future worldwide due to population aging.

**Objective:**

To explore the prevalence of AF in Chinese community population in 2019 and clarify the prevalence of AF complicated with other arrhythmias and myocardial ischemia (MI) events.

**Methods:**

The remote electrocardiogram (ECG) diagnosis system of Xinhua Hospital was assessed to the screen participants with ECG evidence of AF between January 1 and December 31, 2019. The prevalence rates of AF and its association with other arrhythmias and MI events were analyzed and subgroup analysis was performed between different sexes and age groups.

**Results:**

A total of 22,016 AF cases were identified out of all ECGs derived from the remote ECG diagnosis system in 2019. It is estimated that AF was presented in nearly 10.15 million people in China (age-adjusted standardized rate 0.72%, 95% CI 0.20–1.25%) in 2019 and 62% of the AF cases (6.27 million) affected people aged 65 years and above (age-adjusted standardized rate 3.56%, 95% CI 3.28–3.85%). The prevalence rate of AF in males was higher than that in females (*p* < 0.001), and the ventricular rate of AF patients was faster in females (*p* < 0.001) and younger patients (*p* < 0.001). AF patients with lower ventricular rate (under 60 beats per min) were associated with increased prevalence of ventricular escape/escape rhythm [*p* < 0.001, odds ratio (OR) 5.14] and third-degree atrioventricular block (*p* < 0.001, OR 32.05).

**Conclusion:**

The prevalence of AF is higher in the Chinese community population than that was previously reported. AF patients complicated with ECG patterns suggesting myocardial infarction is common in men, and stricter measures should be taken to control the common risk factors of AF and coronary heart disease. It is also important that more attention should be paid to recognize fatal arrhythmias, especially in elderly male patients with AF.

## Introduction

Atrial fibrillation (AF) is the most common sustained arrhythmia in clinic practice, and its incidence is increasing rapidly all over the world (1). AF is associated with an increased risk of stroke, heart failure, cognitive impairment, impaired quality of life, and high medical costs; it eventually increases the risk of all-cause mortality (1, 2).

An epidemiological survey of 29,079 people aged 30–85 in 13 provinces showed that the age-adjusted prevalence rate of AF in China was 0.65% (3). Other two cross-sectional survey of adults (≥35 years old) from general Chinese population in different areas showed that the age-adjusted prevalence of AF in China was 0.7% (4, 5). Also, a community-based survey of 47,841 adults (≥45 years old) in seven geographic regions of China between 2014 and 2016 demonstrated that the weighted AF prevalence was 1.8% (6).

However, due to the small sample size (less than 50,000 people) and limitation of age in the study population, the prevalence of AF in China obtained from these studies lacks of sufficient credibility. At the same time, studies on the relationship between AF and myocardial ischemia (MI) events or other arrhythmis are insufficient. To address these knowledge gaps, we conducted this study, a cross-sectional research, to determine the prevalence of AF and the co-incidence of AF with other MI events or arrhythmias in Chinese community population. We analyzed the differences in the prevalence of AF and other associated arrhythmias or myocardial ischemic events in different population, to supply epidemical evidence, which might be helpful for decision making on controlling the epidemic situation of AF in China.

## Materials and Methods

### Design and Study Population

We conducted a non-randomized, cross-sectional study. Relying on the remote electrocardiogram (ECG) diagnosis system (platform) of the Xinhua Hospital affiliated to Medical College of Shanghai Jiao Tong University, our research covered a total of 1,320 medical institutions in 10 provinces in China. Study population included in this research came from different geographic areas of China, including the Central (Hubei, Jiangxi), the Eastern (Jiangsu, Shanghai), the Northwest (Xinjiang), the Southeast (Guangdong, Zhejiang), and the Southwest (Tibet, Guizhou, Yunnan), and came from both economic developed areas [higher than the national per capita gross domestic product (GDP) level: Shanghai, Jiangsu, Zhejiang, Guangdong, Hubei] and economic less-developed areas (below the national per capita GDP level: Xinjiang, Jiangxi, Xizang, Yunnan, Guizhou) (7). At the same time, the 1320 medical institutions included in this study contained community hospitals and city hospitals.

The remote ECG diagnosis system included all ECG data from those medical institutions, which might derive from outpatient department, emergency department, physical examination center, and so on. We screened all the ECG data from the remote ECG diagnosis system and the total number was 880,149 in 2019, invalid data (*n* = 15,102) were excluded then. For people who underwent multiple ECG examinations, if there was no evidence of atrial fibrillation, the result of the last ECG examination was used, and if one or more ECG examinations indicated atrial fibrillation, the result of the last ECG examination confirming atrial fibrillation was retained. Finally, the results of ECG examination derived from 804,361 participants were analyzed in our study.

### Data Collection and Analysis

All the ECG data were collected by China Food and Drug Administration-certified ECG machines (EDAN SE-1201, MINDRAY BenenHeart R12, NIHON KOHDEN ECG-1250P, and so on), which contained the ECG data of the patient in resting state for 30 s and transmitted automatically after the collection. All ECG data, vector data in digital imaging and communications in medicine format, were encrypted and transmitted through the network and no data were lost in the transmission process.

The demographic characteristics of objects include sex, age, location, and the specific time of ECG examination. After collection and transmission of raw data in the corresponding community hospitals or city hospitals, the invalid data were screened and eliminated by artificial intelligence. Convolutional Neural Network (CNN) was used in ECG artificial intelligence diagnosis. The stability and accuracy of the machine learning algorithm have been validated by the ECG platform. Take the algorithm for AF diagnosis as an example. A deep convolutional residual network (Resnet) was used to stack 15 residual units, and a total of 33 convolution layers were reached. Using rectified linear unit as the activation function, batch normalization and dropout are performed on each layer. Resnet is followed by long short-term memory (LSTM) to summarize the information of each time step. The final output of LSTM is processed by the full connection layer and then Softmax function is used to output the two-dimensional prediction result: atrial fibrillation or not. A total of 100,000 samples were collected as the training set, which contained 10,000 AF and 90,000 non-AF samples. About 5,000 samples, 500 of which were AF, were used as the testing set. There was no intersection between the testing and the training sets. The machine learning algorithm was implemented based on Tensorflow 1.13.1. The hardware environment of the training process was NVIDIA GeForce RTX 2080 Ti and the operating system was Ubuntu 18.04. In the training process, Adam (learning_rate = 0.001) was used as the optimator. The maximum number of iterations was 50, and the loss-function was categorical_crossentropy. When the loss-function could not be further reduced (accuracy was set to 1e-5) or the maximum number of iterations was reached, the training process would be stopped. During the process of training, validation_split was set to 0.2 so that Tensorflow randomly divided 20% of the data as the validation set. After the training, the parameters of CNN were fixed and the data in the testing set was input for prediction. Similar algorithms were adopted in other ECG diagnoses. The machine learning model and details on the training and validation of the machine learning algorithm are shown in Supplementary Figure 1.

Procedures for obtaining the final results of ECG diagnosis are as follows: firstly, the ECG was analyzed by artificial intelligence and preliminary diagnosis was drawn, and then the diagnosis would be reexamined by two doctors. If the diagnosis of two doctors consisted with each other and agreed with artificial intelligence, the diagnosis would be reserved. If two doctors agreed with each other but disagreed with diagnosis from artificial intelligence, the diagnosis results of doctors would be adopted. If one of the doctors’ diagnosis and artificial intelligence diagnosis result were consistent but different from another physician’s, artificial intelligence diagnosis would be adopted. If the two doctors’ diagnosis discorded with each other and both of them were different from artificial intelligence diagnosis, the ECG would be transferred to another physician and the final diagnosis would come from the same two of the four diagnoses above, which was rare in our research. One aim of the current study is to validate the results of machine learning diagnosis, and to see which process is needed to optimize the machine learning diagnosis algorithm. The coincidence rate between two physicians on the same ECG diagnosis was 98% and that was 96% between physicians and machine learning algorithm.

We removed all personally identifiable information to protect personal privacy before analysis.

### Data Classification

Routine 12-lead ECG is one of the main auxiliary examination methods for the diagnosis of MI. We classified all diagnostic information into three categories: normal ECG, ECG patterns suggesting MI, and other abnormal ECG (abnormal ECG without typical MI-related ECG event, which mainly include various arrhythmias). Due to the lack of access to more clinical data, we defined the presence of MI in patients as whether there were abnormal changes related to MI on the ECG according to the Minnesota Code Classification System for Electrocardiographic Findings. These changes mainly included the following types: ECG patterns suggesting MI/ECG-MI (ECG patterns suggesting acute myocardial infarction/ECG-AMI, ECG patterns suggesting subacute myocardial infarction/ECG-subacute MI, ECG patterns suggesting old MI/ECG-old MI, ECG patterns suggesting other MI/ECG-other MI), ECG patterns suggesting suspected MI/ECG-suspected MI (elevated ST segment, abnormal Q wave), and non-specific ST-T abnormality. The specific definitions of ischemic ECG events are shown in Supplementary Table 1.

Also, routine 12-lead electrocardiogram is an important method for the diagnosis of arrhythmias. The diagnosis of additional arrhythmias in participants with AF mainly include the following categories: ventricular extrasystole, ventricular tachycardia, third-degree atrioventricular block/third-degree AVB, intraventricular block (right bundle branch block/RBBB, left bundle branch block/LBBB, left anterior fascicular block, left posterior fascicular block, non-specific intraventricular conduction disturbance), arrhythmias that cannot be definitively diagnosed during the onset of AF were excluded, including atrial extrasystole, atrial tachycardia, first-degree atrioventricular block.

Patients with AF were divided into three groups according to the mean ventricular rate: (1) AF with slow ventricular rate: ventricular rate < 60 bpm; (2) AF with normal ventricular rate: 60 bpm ≤ ventricular rate ≤ 100 bpm; (3) AF with rapid ventricular rate: ventricular rate > 100 bpm.

### Statistical Analysis

We adopted the method of cross-sectional study to investigate the prevalence of AF in Chinese community population. Combined with the results of our research, the data of the 2019 China Population Sampling Survey on Age and Sex Distribution and the estimated population of 1.40005 billion at the end of 2019 (8), we estimated the nationwide prevalence rate of AF in 2019 (the specific process is shown in Supplementary Table 2) and the estimated number of AF patients. The prevalence of all kinds of ECG patterns suggesting MI and arrhythmia events in patients with AF and their distribution in different age and sex groups were analyzed by chi-square test. Also, the distribution of ventricular rate in patients with AF and the prevalence of ECG patterns suggesting MI and other arrhythmia events in patients with different ventricular rate groups were calculated and analyzed using chi-square test and Mann–Whitney *U* test.

In our study, SPSS 22 statistical software was used to analyze the data, and Prism 8 were used to draw the corresponding statistical graphics.

## Results

### Characteristics of the Population

A total of 804,361 valid ECG data across the country were collected during 2019, including 363,695 males (45.22%), 440,666 females (54.78%), 322,772 patients under age of 65 (40.13%), and 481,589 patients aged over 65 years old (59.87%). The specific age distribution characteristics of the population are shown in Table1 and Supplementary Table 3.

Our research focused on the ECG characteristics of patients with AF. A total of 22,106 cases diagnosed with AF by routine 12-lead ECG examination in the remote ECG diagnosis system from all over the country were collected during 2019, which included 11,088 males (50.16%), 11,018 females (49.84%), 20,491 patients aged over 65 years old (92.69%), and 1615 patients under age of 65 (7.31%). The specific age distribution characteristics of the AF population are shown in Table 1 and Supplementary Table 4.

**TABLE 1:** Demographic data of the study population.

	Males, *n* (%)	Females, *n* (%)	Total, *n*
**Age groups of patients**			
<65 years	211853 (43.99)	269736 (56.01)	481589
≥65 years	151842 (47.04)	170930 (52.96)	322772
Total, *n* (%)	363695 (45.22)	440666 (54.78)	804361
**Age groups of patients with AF**			
<65 years	1094 (67.74)	521 (32.26)	1615
≥65 years	9994 (48.77)	10497 (51.23)	20491
Total, *n* (%)	11088 (50.16)	11018 (49.84)	22106

### Prevalence of Atrial Fibrillation

The nationwide prevalence rate of AF in 2019 was about 0.72% (95%CI 0.20–1.25%) and the estimated number of AF patients would reach 10.15 million in 2019. At the same time, the national prevalence rates of AF in men and women in 2019 were estimated to be 0.85% (95% CI 0.10–1.59%) and 0.63% (95% CI −0.12–1.38%), separately. The national prevalence rates of AF in people aged over 65 years and under age of 65 years were 3.56% (95% CI 3.28–3.85%) and 0.32% (95% CI −0.29–0.92%), separately. The specific prevalence of AF in different age groups and sexes is shown in Supplementary Table 5. For investigating the potential influence of COVID-19 pandemic in the prevalence of AF in general population, we also compared the prevalence of AF between December 2019 and January–November 2019, as well as between November–December 2019 and January–October 2019, which showed a higher AF prevalence in the former than that in the latter period, as seen in Table 2.

**TABLE 2:** Comparisons of AF prevalence between different periods in 2019.

Periods	AF		Non-AF		*p* value*
	Number	Percentage	Number	Percentage	
December	1702	3.4%	48015	96.6%	<0.0001
January–November	20404	2.7%	734204	97.3%	
November–December	3209	3.2%	98239	96.8%	<0.0001
January–October	18897	2.7%	684016	97.3%	

*Chi-square test.

### Ventricular Rate in Patients With Atrial Fibrillation

**FIGURE 1:**
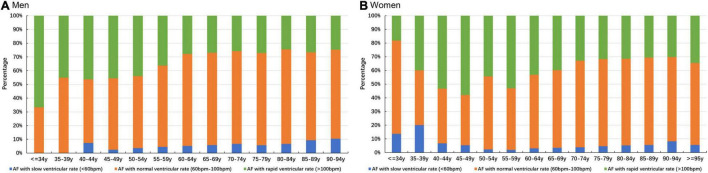
Distribution of ventricular rate in patients with AF. **(A)** Distribution of ventricular rate of male patients with AF. **(B)** Distribution of ventricular rate of female patients with AF.

The distribution of ventricular rate in patients with AF of different ages and sexes is shown in Figure 1 and Supplementary Table 6. The results showed that the ventricular rate was 86 bpm [interquartile range (IQR) 74–103 bpm] and 89 bpm (IQR: 76–108 bpm) separately in male and female patients with AF, and the latter is faster (*p* < 0.001). It also showed that the ventricular rate was 87 bpm (IQR: 74–104 bpm) and 95 bpm (IQR: 80–116 bpm) in older (≥65 years) and younger (<65 years) patients with AF, respectively, and the former is slower (*p* < 0.001). At the same time, we found that after 45 years of age, the proportion of AF with slow and normal ventricular rate increased, while the proportion of AF with rapid ventricular rate decreased with aging in male patients, which can be also observed in females.

In our research, the success rate of ventricular rate control in male patients was higher than that in female patients with AF (*p* < 0.001), which was also higher in elderly patients (*p* < 0.001), no matter in lenient ventricular rate control strategy (resting heart rate < 110 bpm) or in strict ventricular rate control strategy (resting heart rate < 80 bpm). The specific results are shown in Table 3. We also compared AF patients with different control levels of ventricular rate about various MI ECG events, and there was no statistically significant difference, as shown in Supplementary Table 7.

**TABLE 3:** Status of control of ventricular rate in patients with AF.

	<80 bpm			<110 bpm		
	Prevalence rate% (95%CI)	*p* value*	OR (95%CI)^§^	Prevalence rate% (95%CI)	*p* value*	OR (95%CI) ^§^
Males	35.40 (34.51, 36.29)	<0.001	1.19 (1.13, 1.26)	80.90 (80.17, 81.63)	<0.001	1.28 (1.20, 1.37)
Females	31.49 (30.63, 32.36)			76.77 (75.98, 77.55)		
≥65 years	34.19 (33.54, 34.84)	<0.001	1.63 (1.45, 1.84)	79.55 (79.00, 80.10)	<0.001	1.69 (1.51, 1.88)
<65 years	24.15 (22.06, 26.24)			69.78 (67.54, 72.02)		
Total	33.45 (32.83, 34.07)	/	/	78.84 (78.30, 79.38)	/	/

*Chi-square test. ^§^OR, odds ratio.

### Myocardial Ischemia in Patients With Atrial Fibrillation

#### Myocardial Ischemia in Different Sexes of Atrial Fibrillation Patients

**FIGURE 2:**
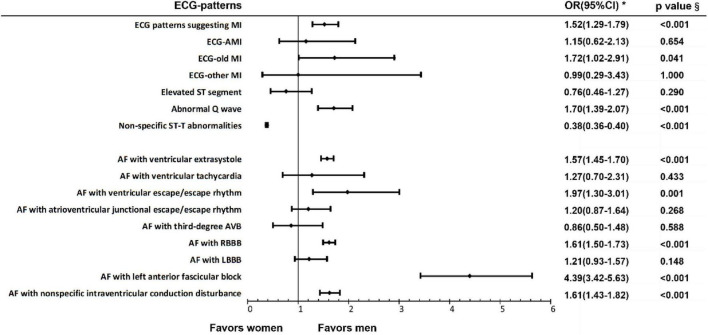
Electrocardiogram patterns suggesting myocardial ischemia and arrhythmias in patients with AF of different sexes. ^§^Chi-square test; *OR, odds ratio.

We found that in patients with AF, the prevalence rate of ECG patterns suggesting MI in men was higher than that in women, and the results was statistically different (OR 1.52, 95% CI 1.29–1.79). According to the classification of various specific ECG patterns suggesting MI, the prevalence rates of ECG-old MI (OR 1.72, 95% CI 1.02–2.91), ECG-suspected MI (OR 1.53, 95% CI 1.28–1.84) and abnormal Q wave (OR 1.70, 95% CI 1.39–2.08) were higher in men. The prevalence rate of non-specific ST-T abnormality (OR 0.38, 95% CI 0.36–0.40) was higher in women. However, the prevalence rates of ECG-AMI, ECG-subacute MI, and elevated ST segment in patients with AF were not significantly different between different sexes. The specific results are shown in Figure 2 and Supplementary Table 8.

#### Myocardial Ischemia Among Different Age Groups of Atrial Fibrillation Patients

**FIGURE 3:**
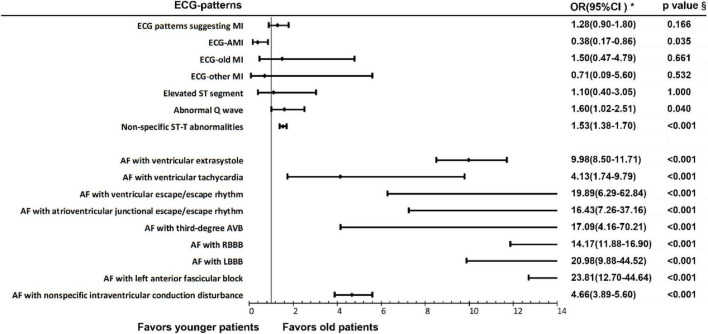
Electrocardiogram patterns suggesting myocardial ischemia and arrhythmias in patients with AF of different age groups. ^§^Chi-square test; *OR, odds ratio.

Our research showed that among patients with AF, the prevalence of ECG patterns suggesting MI in people aged over 65 years old was comparative with that in people under age of 65 (OR 1.28, 95% CI 0.90–1.80). As for the classification of various specific ECG patterns suggesting MI, the prevalence rate of ECG-AMI (2.62, 95% CI 1.16–5.92) was higher in young patients (<65 years old), while the prevalence rate of abnormal Q wave (OR 1.60, 95% CI 1.02–2.51) was higher in older patients (≥65 years old). The prevalence rate of non-specific ST-T abnormality (OR 1.53, 95% CI 1.38–1.70) was higher in older patients (≥65 years old). However, the prevalence rates of ECG-old MI and ECG-subacute MI in patients with AF were not significantly different in older and young patients. The specific results are shown in Figure 3 and Supplementary Table 9.

#### Myocardial Ischemia in Atrial Fibrillation Patients With Different Ventricular Rate

We conducted a further analysis on various ECG patterns suggesting MI in patients diagnosed with AF who manifested different ventricular rate. The results showed that AF with rapid ventricular rate was associated with increased prevalence of ECG-MI (*p* = 0.011) and AF with normal ventricular rate was associated with a decrease in the prevalence of ECG-MI (*p* = 0.010). The specific results are shown in Supplementary Table 10.

### Other Arrhythmias in People With Atrial Fibrillation

#### Other Arrhythmias in Different Sexes of Atrial Fibrillation Patients

We analyzed the prevalence of AF complicated with other arrhythmias in patients of different sexes and the results showed that the prevalence rates of AF with ventricular extrasystole (OR 1.57, 95% CI 1.45–1.70), AF with ventricular escape/escape rhythm (OR 1.97, 95% CI 1.30–3.01), AF with RBBB (OR 1.61, 95% CI 1.50–1.73), AF with left anterior fascicular block (OR 4.39, 95% CI 3.42–5.63) and AF with non-specific intraventricular conduction disturbance (OR 1.61, 95% CI 1.43–1.82) were higher in men. However, there was no significant difference in the prevalence of other arrhythmias such as AF with third-degree AVB in patients with AF between men and women. The specific results are shown in Figure 3 and Supplementary Table 11.

#### Other Arrhythmias in Atrial Fibrillation Patients Among Different Age Groups

The results showed that there were statistical differences in the prevalence of AF complicated with other arrhythmias in patients among different age groups. Specifically, the prevalence rates of AF with ventricular extrasystole (OR 9.98, 95% CI 8.50–11.71), AF with ventricular tachycardia (OR 4.13, 95% CI 1.74–9.79), AF with ventricular escape/escape rhythm (OR 19.89, 95% CI 6.29–62.84), AF with atrioventricular junctional escape/escape rhythm (OR 16.43, 95% CI 7.26–37.16), AF with third-degree AVB (OR 17.09, 95% CI 4.16–70.21), AF with RBBB (OR 14.17, 95% CI 11.86–16.90), AF with LBBB (OR 20.98, 95% CI 9.88–44.52), AF with left anterior fascicular block (OR 23.81, 95% CI 12.70–44.64) and AF with non-specific intraventricular conduction disturbance (OR 4.66, 95% CI 3.89–5.60) were higher in older patients (≥65 years). The specific results are shown in Figure 3 and Supplementary Table 12.

#### Other Arrhythmias in Atrial Fibrillation Patients With Different Ventricular Rate

We conducted a further study focused on other arrhythmic events in patients with AF performing different ventricular rate. The results showed that AF with slow ventricular rate was associated with the increased prevalence of ventricular escape/escape rhythm (OR 5.14, 95% CI 3.42–8.54), atrioventricular junction escape/escape rhythm (OR 16.92, 95%CI 12.25–23.35) and third-degree AVB (OR 32.05, 95% CI 17.79–57.75). AF with rapid ventricular rate was relevant to the declined prevalence of ventricular escape/escape rhythm (OR 0.33, 95%CI 0.18–0.62) and atrioventricular junctional escape/escape rhythm (OR 0.21, 95%CI 0.11–0.37), while AF with normal ventricular rate was related to decreased prevalence of atrioventricular junctional escape/escape rhythm (OR 0.34, 95%CI 0.25–0.48), third-degree AVB (OR 0.24, 95%CI 0.14–0.44). The specific results are shown In Table 4.

**TABLE 4:** Other arrhythmias in AF patients with different ventricular rate.

	AF with slow ventricular rate (<60 bpm)^‡^			AF with normal ventricular rate (60–100 bpm)^‡^			AF with rapid ventricular rate (>100 bpm)^‡^		
	*n* (%)^†^	*p* value^§^	OR (95%CI)*	*n* (%)^†^	*p* value^§^	OR (95%CI)*	*n* (%)^†^	*p* value^§^	OR (95%CI)*
Ventricular extrasystole	124 (8.15)	<0.001	0.67 (0.56, 0.81)	1644 (11.64)	0.257	1.05 (0.96, 1.15)	764 (11.83)	0.257	1.05 (0.96, 1.15)
Ventricular tachycardia	1 (0.07)	0.379	0.32 (0.04, 2.34)	20 (0.14)	0.017	0.49 (0.27, 0.89)	22 (0.34)	0.002	2.54 (1.40, 4.63)
Ventricular escape/escape rhythm	26 (1.71)	<0.001	5.14 (3.42, 8.54)	55 (0.39)	0.409	0.84 (0.55, 1.27)	11 (0.17)	<0.001	0.33 (0.18, 0.62)
Atrioventricular junctional escape/escape rhythm	83 (5.46)	<0.001	16.92 (12.25, 23.35)	58 (0.41)	<0.001	0.34 (0.25, 0.48)	12 (0.19)	<0.001	0.21 (0.11, 0.37)
Third-degree atrioventricular block	37 (2.43)	<0.001	32.05 (17.79, 57.75)	16 (0.11)	<0.001	0.24 (0.14, 0.44)	0	<0.001	/
Intraventricular block	260 (17.09)	0.018	0.85 (0.74, 0.97)	2679 (18.96)	0.026	0.93 (0.86, 0.99)	1351 (20.92)	<0.001	1.14 (1.07, 1.23)
Right bundle branch block	196 (12.89)	0.933	0.99 (0.85, 1.16)	1870 (13.24)	0.099	1.07 (0.99, 1.16)	798 (12.36)	0.089	0.93 (0.85, 1.01)
Left bundle branch block	14 (0.92)	0.682	0.89 (0.52, 1.54)	136 (0.96)	0.240	0.85 (0.65, 1.11)	76 (1.18)	0.142	1.23 (0.93, 1.62)
Left anterior fascicular block	29 (1.91)	0.418	1.17 (0.80, 1.72)	246 (1.74)	0.162	1.17 (0.94, 1.46)	90 (1.39)	0.054	0.79 (0.62, 1.00)
Left posterior fascicular block	1 (0.07)	0.133	13.54 (0.85, 216.611)	0	0.130	/	1 (0.02)	0.499	2.42 (0.15, 38.76)
Non-specific intraventricular conduction disturbance	36 (2.37)	<0.001	0.47 (0.34, 0.66)	563 (3.98)	<0.001	0.64 (0.57, 0.73)	450 (6.97)	<0.001	1.88 (1.66, 2.14)

^‡^Atrial fibrillation patients with heart rate less than 60 bpm were compared with AF patients with heart rate greater than or equal to 60 bpm, AF patients with heart rates ranging from 60 to 100 bpm were compared with other AF patients and AF patients with heart rate greater than or equal to 100 bpm were compared with AF patients with heart rate less than 100 bpm. ^†^The proportion to the number of cases of corresponding ventricular rate categories. ^§^Chi-square test. *OR, odds ratio.

## Discussion

In this large non-randomized, community-based cross-sectional study, we estimated that nearly 10.15 million people in China (0.72% of the population) had a diagnosis of AF based on 12-lead ECG. It should be noticed that 61.79% of the cases (6.27 million) might consist of people aged 65 years and above (3.56% of the population), and 87.00% of the cases (8.83 million) might consist of people aged 45 years and above (1.49% of the population).

Recently published research, a large nationally representative and community-based survey, showed that nearly 8 million adults aged 45 years or more in China (1.8% of the population) had a diagnosis of AF based on either a medical history or a single time-point ECG screen, which was similar to the results of our study. The prevalence results of AF from both of us are significantly higher than that from previous research, 0.65% prevalence of AF in people aged 30–85 years old (3) and 0.71–0.74% in people aged 35 and above (4, 5).

Our research relies on the remote ECG diagnostic platform, which covers a total of 1,320 medical institutions in 10 provinces in China. Thanks to wide coverage of ECG diagnosis platform in China, we research into the population from different geographical areas and different tier of medical institutions in China, covering Central, East, Northwest, Southeast, and Southwest regions, economic developed and underdeveloped areas, city hospitals and community hospitals in China. The number of admissions in this ECG diagnosis platform was on the order of millions for three consecutive years, which is the largest known ECG diagnosis platform in China. Giving all of factors above, we thought population in our study was of good representativeness in Chinese community population even though there is no guarantee of randomized sampling.

### The Prevalence Rate of Atrial Fibrillation Is Higher in Men and Atrial Fibrillation With Myocardial Ischemia Is More Common in Men

It was estimated that there were about 33.6 million patients with AF worldwide by 2010, and there were 21 million men and 12.6 million women with AF (9), which would further increase owing to extended longevity in the general population and intensifying search for undiagnosed AF (10). The age-adjusted incidence, prevalence, and lifetime risk of AF are lower in women vs. men (10, 11). The analysis of people of different sexes in our research shows the same results that the prevalence rate of AF in men is higher than that in women, which agrees with previous research findings in Chinese population (3, 4, 12). Increasing age is a prominent AF risk factor, which also had been proved in our research that the prevalence rate of AF in older patients is higher.

The study period covered COVID-19 pandemic, we therefore compared the prevalence of AF between December 2019 and January–November 2019, as well as between November–December 2019 and January–October 2019. In line with previous reports (13, 14), we also found a higher AF prevalence in the former than that in the latter period, suggesting COVID-19 pandemic might affect the prevalence of AF in general population, future studies are warranted to evaluate the direct impact of COVID-19 on AF in this cohort by comparing the AF prevalence in participants with or without COVID-19 infection. It is also possible that low temperature, high air pollutants, and high sympathetic tension (15, 16) might also contribute to the high AF in winter season, this issue needs to be clarified in future studies.

Previous research suggested that patients with AF had a twofold increased risk of MI (17), and the annual rate of MI in observational studies including AF patients ranged from 0.4 to 2.5% (18). It has also been published that the association between AF and MI was independent of age groups (using 65 or 75 years as a cut point) (17), which was consistent with our results. The REGARDS study elucidates that women are at higher risk of MI compared with men in general population (17). However, our research shows a new result that ECG patterns suggesting MI is more common in men than women in AF population.

### The Ventricular Rate of Patients With Atrial Fibrillation Is Slower in Men and Older Patients

Epidemiological study of status of ventricular rate control in patients with AF is insufficient and it was estimated that 33.58 and 78.76% of patients with AF achieved strict and lenient target of ventricular rate control, respectively, in our research. Rate control is background treatment for nearly all patients with AF, especially for patients who do not require sinus rhythm (e.g., patients aged over 80 years with no or minor symptoms) or for patients with failure of rhythm control (including AF ablation), which also includes patients with new-onset AF and patients with acute recurrences, even if rhythm control is attempted (2, 19). We compared AF patients with different control levels of ventricular rate (resting ventricular rate under 80 bpm or between 80 and 110 bpm) about various MI ECG events, and there was no statistically significant difference, which is consistent with findings from previous research that lenient rate control was non-inferior to strict rate control regarding the development of cardiovascular morbidity and mortality, symptoms, quality of life, and atrial and ventricular remodeling (19, 20). However, due to lack of sufficient clinical information in our research and important disadvantageous effect of faster rates during sinus rhythm (21, 22), we thought that studies with larger sample sizes and longer follow-up periods would be more likely to demonstrate the beneficial effect of stricter ventricular rate control on improved cardiovascular outcomes in patients with AF, which requires further investigation.

### Atrial Fibrillation With Slow Ventricular Rate Is Associated With Increased Prevalence of Escape/Escape Rhythm and Third-Degree Atrioventricular Block

Atrial fibrillation is the most common sustained arrhythmia in clinic, but the research on AF complicated with other arrhythmias is insufficient. Previous studies have shown that sinoatrial node function is impaired in patients with long-term AF, which could lead to clinical manifestations such as sinus arrest and brady–tachy arrhythmia syndrome in patients with AF (23, 24). It has been shown that women’s resting heart rate is higher than men’s (25, 26), which might partially account for that the adverse effect of AF on the function of the sinoatrial node is more pronounced in men. Combined with our research results, it is suggested that more attention should be paid to the monitoring of ventricular rate in male and elderly patients with AF. Also, it is critical to perform early detection in AF patients for finding complicated severe sinus bradycardia or sinus arrest, and early intervention to improve the prognosis.

In addition, research on the occurrence of AF after pacemaker implantation shows that patients with right ventricular pacing (right ventricular apex, right ventricular septum) and dual-chamber pacemaker implantation manifest a higher incidence of AF, while His bundle pacing seems to be associated with a lower risk of persistent or permanent AF (27). On the one hand, it may be more likely to detect silent or asymptomatic AF after pacemaker implantation. On the other hand, studies have shown that bilateral electromechanical asynchrony caused by long-term right ventricular pacing can lead to left ventricular dysfunction and left atrial electromechanical remodeling (28), which are risk factors for AF and these features can also be observed in patients with bundle branch block. However, our research could not clarify the relationship between AF and bundle branch block, which needs further investigation.

## Limitations

At first, due to the insufficient clinical data of participants, we were not able to differentiate patients with complaints of potential cardiovascular diseases, health-seeking subjects with non-cardiovascular diseases, and individuals undergoing routine ECG examination without specific complaints of cardiovascular discomfort. Also, we are unable to classify the type of AF (paroxysmal AF, persistent AF, long-term persistent AF, permanent AF, first diagnosis of AF, non-valvular AF, isolated AF, etc.). Meanwhile, the classification of ECG patterns suggesting MI used in this study is mainly based on the routine 12-lead ECG of patients, and the recording time was 30 s. Although we adopted the definition of ischemic alterations according to the Minnesota Code Classification System for Electrocardiographic Findings (29), it should be noted that classically, a diagnosis of MI requires clinical symptoms and/or myocardial enzyme (troponins, etc.) levels that are suggestive of ischemia, in addition to suggestive ECG signs. An ECG-only classification, without any data of clinical symptom or enzyme levels, is insufficient to reliably classify ischemia.

Second, it is difficult for determining the onset sequence of bundle branch block, AVB, and AF in patients so that we cannot clarify whether there is a causal relationship or interaction between AF and arrhythmias such as AVB and bundle branch block observed in our study.

This research is mainly based on the remote ECG diagnosis platform, which have not yet contained sufficient clinical information such as medical history, medication history, operation history, and so on. Therefore, the success rate of ventricular rate control in patients with AF mentioned in our study only reflects the status of ventricular rate control in AF patients but cannot reflect the influence of various treatment methods. Also, it is difficult for us to analyze the reasons for the differences in the prevalence rate of AF among different populations deeply. Our research team is currently working with the Shanghai Health Commission, hoping to deeply integrate the remote ECG diagnosis platform with the clinical information database for acquiring more clinical information, which is of great significance for further epidemiological analysis of AF and related risk factors, which would be of great importance to define the clinical prognosis of patients with different ECG manifestations.

## Conclusion

It is estimated that the number of patients diagnosed with AF in China would be around 10.15 million in 2019 (0.72% of the population), which would lead to a serious public health burden in the future. Considering the problems in the aging of population, the incidence of AF in the Chinese population will further increase in the future and our findings of a high-prevalence status of AF raise concerns over health policy and practice in this country. In addition, AF patients complicated with ECG patterns suggesting myocardial infarction is common in men, and stricter measures should be taken to control the common risk factors of AF and coronary heart disease. It is also important that more attention should be paid to recognize fatal arrhythmias, especially in elderly male patients with AF.

## Data Availability Statement

The original contributions presented in the study are included in the article/Supplementary Material, further inquiries can be directed to the corresponding author.

## Ethics Statement

The studies involving human participants were reviewed and approved by The Ethics Committee at Xinhua Hospital Affiliated to Shanghai Jiao Tong University School of Medicine. The ethics committee waived the requirement of written informed consent for participation.

## Author Contributions

CL, HaW, and ML analyzed the data, drafted and revised the manuscript, and designed or coded figures and tables. YlZ, BZ, MiC, and XQ developed and maintained the platform of remote ECG diagnosis system. QW, JS, MY, XF, SM, PZ, BL, WL, MuC, YaZ, RZ, and BM critically revised the diagnosis of ECG for further analysis. XL, YeZ, MS, and JH provided critical feedback on data sources. LL provided guidance and support for statistical methods. HoW provided support for the development of remote ECG diagnosis platform. Y-GL designed the study, acquired the fund, and administrated the project. All the authors approved the final version. The corresponding author Y-GL attested that all listed authors meet authorship criteria and that no others meeting the criteria have been omitted.

## Conflict of Interest

XQ, YlZ, BZ, and MiC were employed by the company Shanghai Siwei Medical Co. Ltd. The remaining authors declare that the research was conducted in the absence of any commercial or financial relationships that could be construed as a potential conflict of interest.

## Publisher’s Note

All claims expressed in this article are solely those of the authors and do not necessarily represent those of their affiliated organizations, or those of the publisher, the editors and the reviewers. Any product that may be evaluated in this article, or claim that may be made by its manufacturer, is not guaranteed or endorsed by the publisher.
