# Supplementary material for: Epidemiology of Atrial Fibrillation and Related Myocardial Ischemia or Arrhythmia Events in Chinese Community Population in 2019
Source: Front Cardiovasc Med. 2022 Apr 4;9:821960. doi: 10.3389/fcvm.2022.821960 (PMC9013769; doi:10.3389/fcvm.2022.821960)
Supplement: Supplementary file 1 [file Data_Sheet_1.docx]

**Epidemiology of Atrial Fibrillation and Related Myocardial Ischemia or Arrhythmia Events in Chinese Community Population in 2019**

*Cheng Li^1†^, Haicheng Wang^1†^, Mohan Li^1†^, Xiangjun Qiu^2^, Qunshan Wang^1^, Jian Sun^1^, Mei Yang^1^, Xiangfei Feng^1^, Shu Meng^1^,Pengpai Zhang^1^, Bo Liu^1^, Wei Li^1^, Mu Chen^1^, Yan Zhao^1^, Rui Zhang^1^ ,Binfeng Mo^1^, Yuling Zhu^2^, Baohong Zhou^2^, Min Chen^2^, Xia Liu^3^, Yuelin Zhao^3^, Mingzhen Shen^3^, Jinkang Huang^3^, Li Luo^4^, Hong Wu^5^ and Yi-Gang Li^1,3^*

*^1^ Department of Cardiology, Xinhua Hospital, Shanghai Jiao Tong University School of Medicine, Shanghai, China.*

*^2^ Shanghai Siwei Medical Co. ltd, Shanghai, China.*

*^3^ Medical information telemonitoring center, School of medicine, Shanghai Jiaotong University*

*^4^ School of Public Health, Fudan University, Shanghai, China*

*^5^ Shanghai Municipal Health Commission*

†These authors contributed equally.

■Corresponding Author

Yi-Gang Li, MD, FHRS

Department of Cardiology

Xinhua Hospital, School of Medicine, Shanghai Jiao Tong University

1665 Kongjiang Road, Shanghai, China 200092

E-mail: liyigang@xinhuamed.com.cn

**Supplementary Materials**

Supplementary table 1. Diagnostic criteria for ECG events related to myocardial ischemia.

| Category | Diagnostic criteria | Notes |
| --- | --- | --- |
| AMI | The adjacent leads show the following changes: | Physicians of the remote ECG diagnostic platform would contact with clinicians in hospitals to determine whether the patient complained of chest pain, chest tightness, dyspnea and so on. |
|  | An initial Q wave with a width of 0.03s-0.04s in some leads (MC1-1, MC1-2, MC1-3)  ST segment elevation in leads with Q waves (MC 9-2)  T wave inversion on leads with Q wave (MC 5-1) |  |
| Subacute MI | An initial Q wave with a width of 0.03s-0.04s in some leads (MC1-1, MC1-2, MC1-3)  ST segment fall back to P-R baseline  T wave inversion on leads with Q wave (MC 5-1) |  |
| Old MI | Abnormal Q wave appears in the initial QRS vector (MC1-1, MC1-2, MC1-3)  Abnormal Q waves in adjacent leads  qRs or qrS in leads V1 and V2 | / |
| Elevated ST segment | ST segment elevation in leads with Q waves (MC 9-2) | / |
| Abnormal Q wave | An initial Q wave with a width of 0.03s-0.04s in some leads (MC1-1, MC1-2, MC1-3) | / |
| Non-specific ST-T abnormality | MC4-1, MC 4-2, MC4-3, MC4-4  MC5-1, MC 5-2, MC5-3, MC5-4 | / |

MC, Minnesota Code Classification System for Electrocardiographic Findings

ǂ Prineas R, Crow R, Blackburn H. The Minnesota Code Manual of Electrocardiographic Findings. John Wright-PSG, Inc. Littleton, MA, June 1982.

Supplementary table 2 Data of nationwide census of population in 2010 and 2020 and nationwide sampling survey of population in 2019

|  |  | 2010§ |  |  | 2019* |  |  | 2020ǂ |
| --- | --- | --- | --- | --- | --- | --- | --- | --- |
|  |  | Males, n | Females, n | Total, n(%) | Males, n | Females, n | Total, n(%) | Total, n(%) |
| <15y | | 119794508 | 101528113 | 221322621(16.61) | 98600 | 84667 | 183267(16.78) | 253383938(17.95) |
|  | 0-4y | 41062566 | 34470044 | 75532610(5.67) | 33361 | 29361 | 62722(5.74) | / |
|  | 5-9y | 38464665 | 32416884 | 70881549(5.32) | 32709 | 27992 | 60701(5.56) | / |
|  | 10-14y | 40267277 | 34641185 | 74908462(5.62) | 32530 | 27314 | 59844(5.48) | / |
| 15-59y | | 475494635 | 458399173 | 933893808(70.07) | 363956 | 346657 | 710613(65.08) | 894376020(63.35) |
|  | 15-19y | 51904830 | 47984284 | 99889114(7.49) | 30261 | 25560 | 55821(5.11) | / |
|  | 20-24y | 64008573 | 63403945 | 127412518(9.56) | 32854 | 28665 | 61519(5.63) | / |
|  | 25-29y | 50837038 | 50176814 | 101013852(7.58) | 42186 | 39555 | 81741(7.49) | / |
|  | 30-34y | 49521822 | 47616381 | 97138203(7.29) | 47285 | 46686 | 93971(8.61) | / |
|  | 35-39y | 60391104 | 57634855 | 118025959(8.86) | 39256 | 38447 | 77703(7.12) | / |
|  | 40-44y | 63608678 | 61145286 | 124753964(9.36) | 39234 | 37810 | 77044(7.06) | / |
|  | 45-49y | 53776418 | 51818135 | 105594553(7.92) | 48652 | 46969 | 95621(8.76) | / |
|  | 50-54y | 40363234 | 38389937 | 78753171(5.91) | 47045 | 46079 | 93124(8.53) | / |
|  | 55-59y | 41082938 | 40229536 | 81312474(6.10) | 37183 | 36886 | 74069(6.78) | / |
| 60-64y | | 29834426 | 28832856 | 58667282(4.40) | 30468 | 30244 | 60712(5.56) | 73383486(5.20) |
| >64y | | 57205535 | 61721623 | 118927158(8.92) | 64810 | 72474 | 137284(12.57) | 190635280(13.50) |
|  | 65-69y | 20748471 | 20364811 | 41113282(3.08) | 26967 | 28119 | 55086(5.05) | / |
|  | 70-74y | 16403453 | 16568944 | 32972397(2.47) | 17291 | 18374 | 35665(3.27) | / |
|  | 75-79y | 11278859 | 12573274 | 23852133(1.79) | 10568 | 12042 | 22610(2.07) | / |
|  | 80-84y | 5917502 | 7455696 | 13373198(1.00) | 6242 | 8081 | 14323(1.31) | / |
|  | 85-89y | 2199810 | 3432118 | 5631928(0.42) | 2906 | 4130 | 7036(0.64) | / |
|  | 90-94y | 530872 | 1047435 | 1578307(0.12) | 719 | 1395 | 2114(0.19) | / |
|  | 95y | 126568 | 279345 | 405913(0.03) | 117 | 333 | 450(0.04) | / |
| Overall | | 682329104 | 650481765 | 1332810869(100.00) | 557834 | 534042 | 1091876(100.00) | 1411778724(100.00) |

§ Data of the Population Census in 2010[1].

* Data of nationwide sampling survey of population in 2019[2].

ǂ Data of the Population Census in 2020[3], which have not been disclosed fully yet.

Nationwide census data from the 2010 and 2020 shows that the ratio of men to women (regardless of age) is consistent but there was a significant difference in age constituent ratio between them. However, age constituent ratio was similar between 2019 and 2020.

AF prevalence was estimated with a population weight (age and sex). Population weight was adjusted for deviations in the sample compared with the total adult population in China, according to 2019 China Population Sampling Survey on Age and Sex Distribution, particularly for sex and age[2].

1. Statistics, C.N.B.o. *Data of the Population Census in 2010*. 2011; Available from: <http://www.stats.gov.cn/tjsj/pcsj/rkpc/6rp/indexch.htm>.

2. Statistics, C.N.B.o. *2020 CHINA STATISTICAL YEARBOOK*. 2020; Available from: <http://www.stats.gov.cn/tjsj/ndsj/2020/indexch.htm>.

3. Statistics, C.N.B.o. *Data of the Population Census in 2020*. 2021; Available from: <http://www.stats.gov.cn/tjsj/zxfb/202105/t20210510_1817180.html>.

Supplementary table 3 Demographic data of the study population in total.

| Age groups | Males, n(%) | Females, n(%) | Overall, n |
| --- | --- | --- | --- |
| ≤34y | 42252(48.21) | 45398(51.79) | 87650 |
| 35-39y | 13589(50.71) | 13208(49.29) | 26797 |
| 40-44y | 12715(52.38) | 11561(47.62) | 24276 |
| 45-49y | 13728(50.33) | 13550(49.67) | 27278 |
| 50-54y | 16522(47.90) | 17972(52.10) | 34494 |
| 55-59y | 19220(43.03) | 25446(56.97) | 44666 |
| 60-64y | 33816(43.57) | 43795(56.43) | 77611 |
| 65-69y | 66297(44.50) | 82688(55.50) | 148985 |
| 70-74y | 59162(45.37) | 71244(54.63) | 130406 |
| 75-79y | 37649(44.73) | 46528(55.27) | 84177 |
| 80-84y | 25006(44.23) | 31535(55.77) | 56541 |
| 85-89y | 16760(40.66) | 24464(59.34) | 41224 |
| 90-94y | 5628(35.57) | 10196(64.43) | 15824 |
| ≥95y | 1351(30.48) | 3081(69.52) | 4432 |
| Overall, n(%) | 363695(45.22) | 440666(54.78) | 804361 |

Supplementary table 4 Demographic data of the patients with AF.

| Age groups | Males, n(%) | Females, n(%) | Overall, n |
| --- | --- | --- | --- |
| ≤34y | 31(58.49) | 22(41.51) | 53 |
| 35-39y | 18(64.29) | 10(35.71) | 28 |
| 40-44y | 31(67.39) | 15(32.61) | 46 |
| 45-49y | 54(73.97) | 19(26.03) | 73 |
| 50-54y | 123(73.21) | 45(26.79) | 168 |
| 55-59y | 252(71.19) | 102(28.81) | 354 |
| 60-64y | 585(65.51) | 308(34.49) | 893 |
| 65-69y | 1454(64.42) | 803(35.58) | 2257 |
| 70-74y | 2034(59.02) | 1412(40.98) | 3446 |
| 75-79y | 2016(52.60) | 1817(47.4) | 3833 |
| 80-84y | 1914(47.14) | 2146(52.86) | 4060 |
| 85-89y | 1678(39.99) | 2518(60.01) | 4196 |
| 90-94y | 696(34.08) | 1346(65.92) | 2042 |
| ≥95y | 202(30.75) | 455(69.25) | 657 |
| Overall, n(%) | 11088(50.16) | 11018(49.84) | 22106 |

Supplementary table 5 Prevalence of AF in different age groups.

|  | Overall |  |  | Males |  |  | Females |  |  |
| --- | --- | --- | --- | --- | --- | --- | --- | --- | --- |
|  | Prevalence rate(95%CI) | Standardized rate(95%CI) | Estimated number of cases, million(%)* | Prevalence rate(95%CI) | Standardized rate(95%CI) | Estimated number of cases, million(%)* | Prevalence rate(95%CI) | Standardized rate(95%CI) | Estimated number of cases, million(%)* |
| <35y | 0.06(0.04,0.08) | 0.17(-0.95,1.29) | 1.03(10.13) | 0.07(0.05,0.10) | 0.15(-1.37,1.68) | 0.49(8.18) | 0.05(0.03,0.07) | 0.19(-1.46,1.84) | 0.55(12.65) |
| ≥35y | 3.08(3.04,3.12) | 1.16(0.80,1.51) | 9.12(89.87) | 3.44(3.38,3.50) | 1.41(0.89,1.93) | 5.55(91.82) | 2.78(2.73,2.83) | 0.95(0.45,1.45) | 3.76(87.35) |
| ≥45y | 3.30(3.26,3.34) | 1.49(1.12,1.87) | 8.83(87.00) | 3.73(3.66,3.80) | 1.83(1.27,2.39) | 5.36(88.69) | 2.96(2.91,3.02) | 1.23(0.71,1.74) | 3.66(85.02) |
| ≥55y | 3.60(3.55,3.65) | 2.27(1.94,2.60) | 7.92(78.04) | 4.09(4.01,4.16) | 2.75(2.24,3.26) | 4.67(77.21) | 3.22(3.16,3.28) | 1.92(1.49,2.35) | 3.43(79.63) |
| ≥65y | 4.25(4.20,4.31) | 3.56(3.28,3.85) | 6.27(61.79) | 4.72(4.63,4.81) | 4.05(3.62,4.49) | 3.37(55.70) | 3.89(3.82,3.96) | 3.20(2.81,3.58) | 2.97(68.90) |
| ≥75y | 7.31(7.20,7.43) | 6.69(6.26,7.12) | 3.99(39.34) | 7.53(7.35,7.71) | 7.01(6.35,7.67) | 1.85(30.55) | 7.15(7.00,7.30) | 6.46(5.89,7.03) | 2.15(49.94) |
| ≥85y | 11.22(10.97,11.46) | 11.00(10.24,11.75) | 1.35(13.34) | 10.85(10.46,11.25) | 10.62(9.40,11.84) | 0.51(8.42) | 11.44(11.12,11.76) | 11.24(10.28,12.20) | 0.84(19.59) |
| Overall | 2.75(2.71,2.78) | 0.72(0.20,1.25) | 10.15(100.00) | 3.05(2.99,3.10) | 0.85(0.10,1.59) | 6.05(100.00) | 2.50(2.45,2.55) | 0.63(-0.12,1.38) | 4.31(100.00) |

* Percentage of all patients with AF.

Supplementary table 6 Ventricular rate in patients with AF

|  | | Males, median(IQR) * | Females, median(IQR) | p value ¶ | Overall, median(IQR) |
| --- | --- | --- | --- | --- | --- |
| Age-Groups | |  |  |  |  |
| <65y | | 94(80-114) | 97(80-121) | 0.084 | 95(80-115) |
|  | ≤34y | 100(88-118) | 84.5(71-96) | 0.009 | 93(79-110.5) |
|  | 35-39y | 116(92-130) | 95.5(77-120) | 0.144 | 111.5(87.5-129.5) |
|  | 40-44y | 99(79-118) | 102(94.5-125) | 0.534 | 99.5(81-123) |
|  | 45-49y | 96(81-123) | 111(85.5-139) | 0.250 | 99(81-127) |
|  | 50-54y | 97(84.5-118) | 99(79-124) | 0.871 | 98(84-119.5) |
|  | 55-59y | 98(82-116.5) | 101.5(81-131) | 0.277 | 98.5(82-120) |
|  | 60-64y | 92(77-109) | 95.5(80.5-116) | 0.014 | 93(78-112) |
| ≥65y | | 86(73-102) | 89(76-107) | <0.001 | 87(74-104) |
|  | 65-69y | 87(75-102) | 94(80-112.5) | <0.001 | 89(77-106) |
|  | 70-74y | 87(74-102) | 90(77-108.5) | <0.001 | 88(75-104) |
|  | 75-79y | 86(74-101) | 90(76-106) | <0.001 | 87(75-104) |
|  | 80-84y | 86(73-103) | 88(75-106) | 0.003 | 87(74-105) |
|  | 85-89y | 84(72-100) | 88(75-106) | <0.001 | 86(74-104) |
|  | 90-94y | 84(71-102) | 86(73-106) | 0.021 | 85.5(72-104) |
|  | ≥95y | 85(67-100) | 91(76-111) | 0.001 | 87(73-109) |
| Total, median(IQR) | | 86(74-103) | 89(76-108) | <0.001 | 88(75-105) |

* IQR=Interquartile range

¶ All comparisons were compared by Mann-Whitney test.

Supplementary table 7. Differences in myocardial ischemic ECG events under different ventricular rate controls

|  | <80bpm | | 80-110bpm | | p* | OR(95%CI)§ |
| --- | --- | --- | --- | --- | --- | --- |
|  | n | Prevalence rate(95%CI) | n | Prevalence rate(95%CI) |  |  |
| MI-related ECG events | 247 | 3.34(2.93,3.75) | 227 | 2.26(1.97,2.55) | <0.001 | 1.49(1.24,1.79) |
| MI | 40 | 0.54(0.37,0.71) | 46 | 0.46(0.33,0.59) | 0.443 | 1.18(0.77,1.81) |
| Acute MI | 13 | 0.18(0.08,0.27) | 16 | 0.16(0.08,0.24) | 0.794 | 1.10(0.53,2.29) |
| Subacute MI | 0 | 0 | 3 | 0.03(0.00,0.06) | 0.367 | / |
| Old MI | 21 | 0.28(0.16,0.41) | 26 | 0.26(0.16,0.36) | 0.755 | 1.10(0.62,1.95) |
| Other MI | 6 | 0.08(0.02,0.15) | 1 | 0.01(-0.01,0.03) | 0.053 | 8.15(0.98,67.68) |
| Suspected MI | 207 | 2.80(2.42,3.18) | 181 | 1.80(1.54,2.06) | <0.001 | 1.57(1.28,1.92) |
| Elevated ST segment | 24 | 0.32(0.19,0.45) | 18 | 0.18(0.10,0.26) | 0.053 | 1.81(0.98,3.34) |
| Abnormal Q wave | 183 | 2.47(2.12,2.83) | 163 | 1.62(1.38,1.87) | <0.001 | 1.54(1.24,1.90) |
| Non-specific ST-T abnormality | 3626 | 49.03(47.89,50.17) | 5035 | 50.18(49.21,51.16) | 0.133 | 0.95(0.90,1.01) |
| ST segment depression | 985 | 13.32(12.55,14.09) | 1288 | 12.84(12.18,13.49) | 0.350 | 1.04(0.95,1.14) |
| Inverted T wave | 850 | 11.49(10.77,12.22) | 1004 | 10.01(9.42,10.59) | 0.002 | 1.17(1.06,1.29) |
| Other ST-T changes | 1791 | 24.22(23.24,25.20) | 2743 | 27.34(26.47,28.21) | <0.001 | 0.85(0.79,0.91) |

* Chi-square test

§ OR=odds ratio

Supplementary table 8. Myocardial ischemia in AF patients of different sexes.

|  | Males, n(%) | Females, n(%) | Total, n | p value § | OR (95%CI) * |
| --- | --- | --- | --- | --- | --- |
| MI-related ECG events | 360(3.25) | 238(2.16) | 598 | <0.001 | 1.52(1.29,1.79) |
| MI | 68(0.61) | 47(0.43) | 115 | 0.054 | 1.44(0.99,2.09) |
| Acute MI | 22(0.20) | 19(0.17) | 41 | 0.654 | 1.15(0.62,2.13) |
| Subacute MI | 3(0.03) | 1(0.01) | 4 | 0.622 | 2.98(0.31,28.67) |
| Old MI | 38(0.34) | 22(0.20) | 60 | 0.041 | 1.72(1.02,2.91) |
| Other MI | 5(0.05) | 5(0.05) | 10 | 1.000 | 0.99(0.29,3.43) |
| Suspected MI | 292(2.63) | 191(1.73) | 483 | <0.001 | 1.53(1.28,1.84) |
| Elevated ST segment | 26(0.23) | 34(0.31) | 60 | 0.290 | 0.76(0.46,1.27) |
| Abnormal Q wave | 266(2.40) | 157(1.42) | 423 | <0.001 | 1.70(1.39,2.08) |
| Non-specific ST-T abnormality | 4290(38.69) | 6887(62.51) | 11177 | <0.001 | 0.38(0.36,0.40) |
| ST segment depression | 1164(10.50) | 1986(18.03) | 3150 | <0.001 | 0.53(0.49,0.58) |
| Inverted T wave | 739(6.66) | 1364(12.38) | 2103 | <0.001 | 0.51(0.46,0.56) |
| Other ST-T changes | 2387(21.53) | 3537(32.10) | 5924 | <0.001 | 0.58(0.55,0.62) |

§ Chi-square test

* OR, odds ratio

Supplementary table 9. Myocardial ischemia in AF patients of different age groups.

|  | ≥65y, n(%) | <65y, n(%) | Total, n | p value § | OR (95%CI) * |
| --- | --- | --- | --- | --- | --- |
| MI-related ECG events | 563(2.75) | 35(2.17) | 598 | 0.166 | 1.28(0.90,1.80) |
| MI | 104(0.51) | 11(0.68) | 115 | 0.351 | 0.74(0.40,1.39) |
| Acute MI | 34(0.17) | 7(0.43) | 41 | 0.035 | 0.38(0.17,0.86) |
| Subacute MI | 4(0.02) | 0 | 4 | 1.000 | / |
| Old MI | 57(0.28) | 3(0.19) | 60 | 0.661 | 1.50(0.47,4.79) |
| Other MI | 9(0.04) | 1(0.06) | 10 | 0.532 | 0.71(0.09,5.60) |
| Suspected MI | 459(2.24) | 24(1.49) | 483 | 0.046 | 1.52(1.00,2.30) |
| Elevated ST segment | 56(0.27) | 4(0.25) | 60 | 1.000 | 1.10(0.40,3.05) |
| Abnormal Q wave | 403(1.97) | 20(1.24) | 423 | 0.040 | 1.60(1.02,2.51) |
| Non-specific ST-T abnormality | 10519(51.33) | 658(40.74) | 11177 | <0.001 | 1.53(1.38,1.70) |
| ST segment depression | 2949(14.39) | 201(12.45) | 3150 | 0.031 | 1.18(1.02,1.38) |
| Inverted T wave | 2007(9.79) | 96(5.94) | 2103 | <0.001 | 1.72(1.39,2.12) |
| Other ST-T changes | 5563(27.15) | 361(22.35) | 5924 | <0.001 | 1.29(1.15,1.46) |

§ Chi-square test

* OR, odds ratio

Supplementary table 10. Myocardial ischemia in AF patients with different ventricular rate.

|  | AF with slow ventricular rate (<60bpm)ǂ | | | AF with normal ventricular rate (60bpm-100bpm)ǂ | | | AF with rapid ventricular rate (>100bpm)ǂ | | |
| --- | --- | --- | --- | --- | --- | --- | --- | --- | --- |
|  | n (%) ƚ | p value § | OR (95%CI) * | n (%) ƚ | p value § | OR (95%CI) * | n (%) ƚ | p value § | OR (95%CI) * |
| MI-related ECG events | 46(3.02) | 0.427 | 1.13(0.83,1.54) | 374(2.65) | 0.480 | 0.94(0.80,1.11) | 178 (2.76) | 0.762 | 1.03(0.86,1.23) |
| MI | 8(0.53) | 0.974 | 1.01(0.49,2.08) | 61(0.43) | 0.015 | 0.64(0.44,0.92) | 46(0.71) | 0.011 | 1.62(1.11,2.36) |
| Acute MI | 1(0.07) | 0.415 | 0.34(0.05,2.46) | 23(0.16) | 0.297 | 0.72(0.39,1.34) | 17(0.26) | 0.084 | 1.72(0.92,3.20) |
| Subacute MI | 0 | 1.000 | / | 3(0.02) | 1.000 | 1.69(0.18,16.29) | 1(0.02) | 1.000 | 0.81(0.08,7.77) |
| Old MI | 6(0.39) | 0.484 | 1.51(0.65,3.51) | 31(0.22) | 0.048 | 0.60(0.36,1.00) | 23(0.36) | 0.120 | 1.51(0.90,2.54) |
| Other MI | 1(0.07) | 0.510 | 1.50(0.19,11.88) | 4(0.03) | 0.213 | 0.38(0.11,1.33) | 5(0.08) | 0.272 | 2.43(0.70,8.38) |
| Suspected MI | 38(2.50) | 0.386 | 1.16(0.83,1.62) | 313(2.22) | 0.679 | 1.04(0.86,1.26) | 132(2.04) | 0.358 | 0.91(0.74,1.11) |
| Elevated ST segment | 4(0.26) | 1.000 | 0.97(0.35,2.67) | 41(0.29) | 0.475 | 1.22(0.71,2.10) | 15(0.23) | 0.473 | 0.81(0.45,1.45) |
| Abnormal Q wave | 34(2.24) | 0.342 | 1.19(0.83,1.69) | 272(1.93) | 0.865 | 1.02(0.83,1.24) | 117(1.81) | 0.479 | 0.93(0.75,1.15) |
| Non-specific ST-T abnormality | 730(47.99) | 0.038 | 0.90(0.81,0.99) | 7106(50.30) | 0.297 | 0.97(0.92,1.03) | 3341(51.74) | 0.024 | 1.07(1.01,1.13) |
| ST segment depression | 213(14.00) | 0.776 | 0.98(0.84,1.14) | 1884(13.34) | <0.001 | 0.82(0.76,0.88) | 1053(16.31) | <0.001 | 1.26(1.16,1.37) |
| Inverted T wave | 179(11.77) | 0.002 | 1.29(1.10,1.52) | 1493(10.57) | <0.001 | 1.43(1.29,1.58) | 431(6.67) | <0.001 | 0.60(0.54,0.67) |
| Other ST-T changes | 338(22.22) | <0.001 | 0.77(0.68,0.87) | 3729(26.39) | 0.071 | 0.95(0.89,1.01) | 1857(28.76) | <0.001 | 1.15(1.08,1.23) |

ǂ AF patients with heart rate less than 60bpm were compared with AF patients with heart rate greater than or equal to 60bpm, AF patients with heart rates ranging from 60 to 100bpm were compared with other AF patients and AF patients with heart rate greater than or equal to 100bpm were compared with AF patients with heart rate less than 100bpm.

ƚ The proportion to the number of cases of corresponding ventricular rate categories

§ Chi-square test

* OR, odds ratio

Supplementary table 11. Other arrhythmias in AF patients of different sexes.

|  | Males, n(%) | Females, n(%) | Total, n | p value § | OR (95%CI) * |
| --- | --- | --- | --- | --- | --- |
| AF with ventricular extrasystole | 1429(0.39) | 1103(0.25) | 2532 | <0.001 | 1.57(1.45,1.70) |
| AF with ventricular tachycardia | 22(0.01) | 21(<0.01) | 43 | 0.433 | 1.27(0.70,2.31) |
| AF with ventricular escape/escape rhythm | 57(0.02) | 35(0.01) | 92 | 0.001 | 1.97(1.30,3.01) |
| AF with atrioventricular junctional escape/escape rhythm | 76(0.02) | 77(0.02) | 153 | 0.268 | 1.20(0.87,1.64) |
| AF with third-degree atrioventricular block | 22(0.01) | 31(0.01) | 53 | 0.588 | 0.86(0.50,1.49) |
| AF with intraventricular block | 2470(0.68) | 1820(0.41) | 4290 | <0.001 | 1.65(1.55,1.75) |
| AF with right bundle branch block | 1633(0.45) | 1231(0.28) | 2864 | <0.001 | 1.61(1.50,1.73) |
| AF with left bundle branch block | 113(0.03) | 113(0.03) | 226 | 0.148 | 1.21(0.93,1.57) |
| AF with left anterior fascicular block | 286(0.08) | 79(0.02) | 365 | <0.001 | 4.39(3.42,5.63) |
| AF with left posterior fascicular block | 2(<0.01) | 0 | 2 | 0.204 | / |
| AF with nonspecific intraventricular conduction disturbance | 599(0.16) | 450(0.10) | 1049 | <0.001 | 1.61(1.43,1.82) |

§ Chi-square test

* OR, odds ratio

Supplementary table 12. Other arrhythmias in AF patients of different age groups.

|  | ≥65y, n(%) | <65y, n(%) | Total, n | p value§ | OR (95%CI) * |
| --- | --- | --- | --- | --- | --- |
| AF with ventricular extrasystole | 2372(0.49) | 160(0.05) | 2532 | <0.001 | 9.98(8.50,11.71) |
| AF with ventricular tachycardia | 37(0.01) | 6(<0.01) | 43 | <0.001 | 4.13(1.74,9.79) |
| AF with ventricular escape/escape rhythm | 89(0.02) | 3(<0.01) | 92 | <0.001 | 19.89(6.29,62.84) |
| AF with atrioventricular junctional escape/escape rhythm | 147(0.03) | 6(<0.01) | 153 | <0.001 | 16.43(7.26,37.16) |
| AF with third-degree atrioventricular block | 51(0.01) | 2(<0.01) | 53 | <0.001 | 17.09(4.16,70.21) |
| AF with intraventricular block | 4024(0.84) | 266(0.08) | 4290 | <0.001 | 10.22(9.02,11.57) |
| AF with right bundle branch block | 2734(0.57) | 130(0.04) | 2864 | <0.001 | 14.17(11.88,16.90) |
| AF with left bundle branch block | 219(0.05) | 7(<0.01) | 226 | <0.001 | 20.98(9.88,44.52) |
| AF with left anterior fascicular block | 355(0.07) | 10(<0.01) | 365 | <0.001 | 23.81(12.70,44.64) |
| AF with left posterior fascicular block | 2(<0.01) | 0 | 2 | 0.519 | / |
| AF with nonspecific intraventricular conduction disturbance | 917(0.19) | 132(0.04) | 1049 | <0.001 | 4.66(3.89,5.60) |

§ Chi-square test

* OR, odds ratio

Supplementary table 13. The list of the brand and model of the electrocardiogram equipment.

| Brand | Model |
| --- | --- |
| EDAN | SE-1200Express |
|  | SE-1201 |
|  | SE-1202 |
|  | SE-12Express |
|  | SE-18 |
|  | SE-300A |
|  | SE-300B |
|  | SE-301 |
|  | SE-3A |
|  | SE-3B |
|  | SE-601A |
|  | SE-601B |
|  | SE-601C |
| MINDRAY | BenenHeart R3 |
|  | BenenHeart R12 |
| NIHON KOHDEN | ECG-1250P |
|  | ECG-2110 |
|  | ECG-2150 |
|  | ECG-2250 |
|  | ECG-2260 |
|  | ECG-2340/2350 |
|  | ECG-2360 |

Supplementary figure 1. The machine learning model and details on the training and validation of the machine learning algorithm


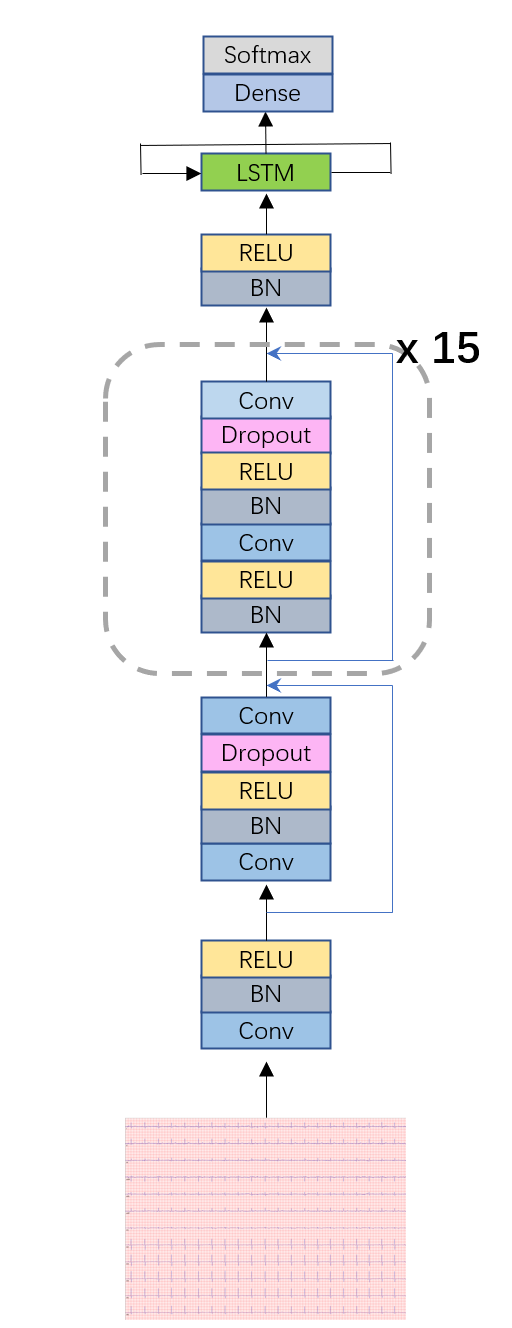


|  | | Model prediction | |
| --- | --- | --- | --- |
|  |  | AF | Non-AF |
| Ground truth | AF | 467 | 33 |
|  | Non-AF | 68 | 4432 |

|  | Recall | Precision | F-Measure |
| --- | --- | --- | --- |
| AF | 0.934 | 0.872 | 0.91 |
| Non-AF | 0.985 | 0.993 | 0.99 |
